# Turnover intention and its associated factors among nurses in Ethiopia: a systematic review and meta-analysis

**DOI:** 10.1186/s12913-024-11122-9

**Published:** 2024-05-24

**Authors:** Eshetu Elfios, Israel Asale, Merid Merkine, Temesgen Geta, Kidist Ashager, Getachew Nigussie, Ayele Agena, Bizuayehu Atinafu, Eskindir Israel, Teketel Tesfaye

**Affiliations:** 1 School of Nursing, College of Health Science and Medicine, Wolaita Sodo University, Wolaita Sodo, Ethiopia; 2 Department of Midwifery, College of Health Science and Medicine, Wolaita Sodo University, Wolaita Sodo, Ethiopia; 3 Department of Midwifery, College of Health Science and Medicine, Wachamo University, Hossana, Ethiopia

**Keywords:** Turnover intention, Nurses, Systematic review, Meta-analysis, Ethiopia

## Abstract

**Background:**

Nurses turnover intention, representing the extent to which nurses express a desire to leave their current positions, is a critical global public health challenge. This issue significantly affects the healthcare workforce, contributing to disruptions in healthcare delivery and organizational stability. In Ethiopia, a country facing its own unique set of healthcare challenges, understanding and mitigating nursing turnover are of paramount importance. Hence, the objectives of this systematic review and meta-analysis were to determine the pooled proportion ofturnover intention among nurses and to identify factors associated to it in Ethiopia.

**Methods:**

A comprehensive search carried out for studies with full document and written in English language through an electronic web-based search strategy from databases including PubMed, CINAHL, Cochrane Library, Embase, Google Scholar and Ethiopian University Repository online. Checklist from the Joanna Briggs Institute (JBI) was used to assess the studies’ quality. STATA version 17 software was used for statistical analyses. Meta-analysis was done using a random-effects method. Heterogeneity between the primary studies was assessed by Cochran Q and I-square tests. Subgroup and sensitivity analyses were carried out to clarify the source of heterogeneity.

**Result:**

This systematic review and meta-analysis incorporated 8 articles, involving 3033 nurses in the analysis. The pooled proportion of turnover intention among nurses in Ethiopia was 53.35% (95% CI (41.64, 65.05%)), with significant heterogeneity between studies (I^2^ = 97.9, *P* = 0.001). Significant association of turnover intention among nurses was found with autonomous decision-making (OR: 0.28, CI: 0.14, 0.70) and promotion/development (OR: 0.67, C.I: 0.46, 0.89).

**Conclusion and recommendation:**

Our meta-analysis on turnover intention among Ethiopian nurses highlights a significant challenge, with a pooled proportion of 53.35%. Regional variations, such as the highest turnover in Addis Ababa and the lowest in Sidama, underscore the need for tailored interventions. The findings reveal a strong link between turnover intention and factors like autonomous decision-making and promotion/development. Recommendations for stakeholders and concerned bodies involve formulating targeted retention strategies, addressing regional variations, collaborating for nurse welfare advocacy, prioritizing career advancement, reviewing policies for nurse retention improvement.

## Background

Turnover intention pertaining to employment, often referred to as the intention to leave, is characterized by an employee’s contemplation of voluntarily transitioning to a different job or company [1]. Nurse turnover intention, representing the extent to which nurses express a desire to leave their current positions, is a critical global public health challenge. This issue significantly affects the healthcare workforce, contributing to disruptions in healthcare delivery and organizational stability [2].

The global shortage of healthcare professionals, including nurses, is an ongoing challenge that significantly impacts the capacity of healthcare systems to provide quality services [3]. Nurses, as frontline healthcare providers, play a central role in patient care, making their retention crucial for maintaining the functionality and effectiveness of healthcare delivery. However, the phenomenon of turnover intention, reflecting a nurse’s contemplation of leaving their profession, poses a serious threat to workforce stability [4].

Studies conducted globally shows that high turnover rates among nurses in several regions, with notable figures reported in Alexandria (68%), China (63.88%), and Jordan (60.9%) [5–7]. In contrast, Israel has a remarkably low turnover rate of9% [8], while Brazil reports 21.1% [9], and Saudi hospitals26% [10]. These diverse turnover rates highlight the global nature of the nurse turnover phenomenon, indicating varying degrees of workforce mobility in different regions.

The magnitude and severity of turnover intention among nurses worldwide underscore the urgency of addressing this issue. High turnover rates not only disrupt healthcare services but also result in a loss of valuable skills and expertise within the nursing workforce. This, in turn, compromises the continuity and quality of patient care, with potential implications for patient outcomes and overall health service delivery [11]. Extensive research conducted worldwide has identified a range of factors contributing to turnover intention among nurses [11–17]. These factors encompass both individual and organizational aspects, such as high workload, inadequate support, limited career advancement opportunities, job satisfaction, conflict, payment or reward, burnout sense of belongingness to their work environment. The complex interplay of these factors makes addressing turnover intention a multifaceted challenge that requires targeted interventions.

In Ethiopia, a country facing its own unique set of healthcare challenges, understanding and mitigating nursing turnover are of paramount importance. The healthcare system in Ethiopia grapples with issues like resource constraints, infrastructural limitations, and disparities in healthcare access [18]. Consequently, the factors influencing nursing turnover in Ethiopia may differ from those in other regions. Previous studies conducted in the Ethiopian context have started to unravel some of these factors, emphasizing the need for a more comprehensive examination [18, 19].

Although many cross-sectional studies have been conducted on turnover intention among nurses in Ethiopia, the results exhibit variations. The reported turnover intention rates range from a minimum of 30.6% to a maximum of 80.6%. In light of these disparities, this systematic review and meta-analysis was undertaken to ascertain the aggregated prevalence of turnover intention among nurses in Ethiopia. By systematically analyzing findings from various studies, we aimed to provide a nuanced understanding of the factors influencing turnover intention specific to the Ethiopian healthcare context. Therefore, this systematic review and meta-analysis aimed to answer the following research questions.

1. What is the pooled prevalence of turnover intention among nurses in Ethiopia?
2. What are the factors associated with turnover intention among nurses in Ethiopia?

### Objectives

The primary objective of this review was to assess the pooled proportion of turnover intention among nurses in Ethiopia. The secondary objective was identifying the factors associated to turnover intention among nurses in Ethiopia.

## Methods

### Study design and search strategy

A comprehensive systematic review and meta-analysis was conducted, examining observational studies on turnover intention among nurses in Ethiopia. The procedure for this systematic review and meta-analysis was developed in accordance with the Preferred Reporting Items for Systematic review and Meta-analysis Protocols (PRISMA-P) statement [20]. PRISMA-2015 statement was used to report the findings [21, 22]. This systematic review and meta-analysis were registered on PROSPERO with the registration number of CRD42024499119.

We conducted systematic and an extensive search across multiple databases, including PubMed, CINAHL, Cochrane Library, Embase, Google Scholar and Ethiopian University Repository online to identify studies reporting turnover intention among nurses in Ethiopia. We reviewed the database available at http://www.library.ucsf.edu and the Cochrane Library to ensure that the intended task had not been previously undertaken, preventing any duplication. Furthermore, we screened the reference lists to retrieve relevant articles. The process involved utilizing EndNote (version X8) software for downloading, organizing, reviewing, and citing articles. Additionally, a manual search for cross-references was performed to discover any relevant studies not captured through the initial database search. The search employed a comprehensive set of the following search terms:“prevalence”, “turnover intention”, “intention to leave”, “attrition”, “employee attrition”, “nursing staff turnover”, “Ethiopian nurses”, “nurses”, and “Ethiopia”. These terms were combined using Boolean operators (AND, OR) to conduct a thorough and systematic search across the specified databases.

### Eligibility criteria

#### Inclusion criteria

The established inclusion criteria for this meta-analysis and systematic review are as follows to guide the selection of articles for inclusion in this review.

1. Population: Nurses working in Ethiopia.
2. Study period: studies conducted or published until 23November 2023.
3. Study design: All observational study designs, such as cross-sectional, longitudinal, and cohort studies, were considered.
4. Setting: Only studies conducted in Ethiopia were included.
5. Outcome; turnover intention.
6. Study: All studies, whether published or unpublished, in the form of journal articles, master’s theses, and dissertations, were included up to the final date of data analysis.
7. Language: This study exclusively considered studies in the English language.

#### Exclusion criteria

Excluded were studies lacking full text or Studies with a Newcastle–Ottawa Quality Assessment Scale (NOS) score of 6 or less. Studies failing to provide information on turnover intention among nurses or studies for which necessary details could not be obtained were excluded. Three authors (E.E., T.G., K.A) independently assessed the eligibility of retrieved studies, other two authors (E.I & M.M) input sought for consensus on potential in- or exclusion.

### Quality assessment and data extraction

Two authors (E.E, A.A, G.N) independently conducted a critical appraisal of the included studies. Joanna Briggs Institute (JBI) checklists of prevalence study was used to assess the quality of the studies. Studies with a Newcastle–Ottawa Quality Assessment Scale (NOS) score of seven or more were considered acceptable [23]. The tool has nine parameters, which have yes, no, unclear, and not applicable options [24]. Two reviewers (I.A, B.A) were involved when necessary, during the critical appraisal process. Accordingly, all studies were included in our review. **(**Table 1**)**Questions to evaluate the methodological quality of studies on turnover intention among nurses and its associated factors in Ethiopia are the followings:

Q1 = was the sample frame appropriate to address the target population?

Q2. Were study participants sampled appropriately.

Q3. Was the sample size adequate?

Q4. Were the study subjects and the setting described in detail?

Q5. Was the data analysis conducted with sufficient coverage of the identified sample?

Q6. Were the valid methods used for the identification of the condition?

Q7. Was the condition measured in a standard, reliable way for all participants?

Q8. Was there appropriate statistical analysis?

Q9. Was the response rate adequate, and if not, was the low response rate.

managed appropriately?

**Table 1:** Critical appraisal results of eligible studies in this study on turnover intention among nurses and associated factors, Ethiopia, 2024

Name of author	Q1	Q2	Q3	Q4	Q5	Q6	Q7	Q8	Q9	Total
Gebregziabher D	Y	Y	Y	Y	Y	Y	Y	Y	Y	9
Asegid A et al.	Y	Y	Y	Y	Y	Y	Y	Y	Y	9
Getie G.A et al.	Y	Y	Y	Y	Y	Y	Y	Y	Y	9
Ayalew F et al.	Y	Y	Y	Y	Y	Y	Y	Y	Y	9
Getachew N et al.	Y	Y	Y	Y	Y	N	Y	Y	Y	8
Wubetie A et al.	Y	Y	Y	Y	Y	Y	Y	Y	Y	9
Fekadu et al.	Y	N	Y	Y	Y	Y	Y	Y	Y	8
Negarandeh R et al.	Y	Y	Y	Y	Y	Y	N	Y	Y	8

*Y* Yes, *N* No, JBI critical appraisal checklist for studies reporting prevalence data: Q1 = was the sample frame appropriate to address the target population? Q2-Were study participants sampled appropriately? Q3-Was the sample size adequate? Q4-Were the study subjects and the setting described in detail? Q5-Was the data analysis conducted with sufficient coverage of the identified sample. Q6-Were the valid methods used for the identification of the condition? Q7-Was the condition measured in a standard, reliable way for all participants? Q8-Was there appropriate statistical analysis? Q9-Was the response rate adequate, and if not, was the low response rate managed appropriately?

Data was extracted and recorded in a Microsoft Excel as guided by the Joanna Briggs Institute (JBI) data extraction form for observational studies. Three authors (E.E, M.G, T.T) independently conducted data extraction. Recorded data included the first author’s last name, publication year, study setting or country, region, study design, study period, sample size, response rate, population, type of management, proportion of turnover intention, and associated factors. Discrepancies in data extraction were resolved through discussion between extractors.

### Data processing and analysis

Data analysis procedures involved importing the extracted data into STATA 14 statistical software for conducting a pooled proportion of turnover intention among nurses. To evaluate potential publication bias and small study effects, both funnel plots and Egger’s test were employed [25, 26]. We used statistical tests such as the I statistic to quantify heterogeneity and explore potential sources of variability. Additionally, subgroup analyses were conducted to investigate the impact of specific study characteristics on the overall results. I^2^values of 0%, 25%, 50%, and 75% were interpreted as indicating no, low, medium, and high heterogeneity, respectively [27].

To assess publication bias, we employed several methods, including funnel plots and Egger’s test. These techniques allowed us to visually inspect asymmetry in the distribution of study results and statistically evaluate the presence of publication bias. Furthermore, we conducted sensitivity analyses to assess the robustness of our findings to potential publication bias and other sources of bias.

Utilizing a random-effects method, a meta-analysis was performed to assess turnover intention among nurses, employing this method to account for observed variability [28]. Subgroup analyses were conducted to compare the pooled magnitude of turnover intention among nurses and associated factors across different regions. The results of the pooled prevalence were visually presented in a forest plot format with a 95% confidence interval.

## Result

### Study selection

**Fig. 1:**
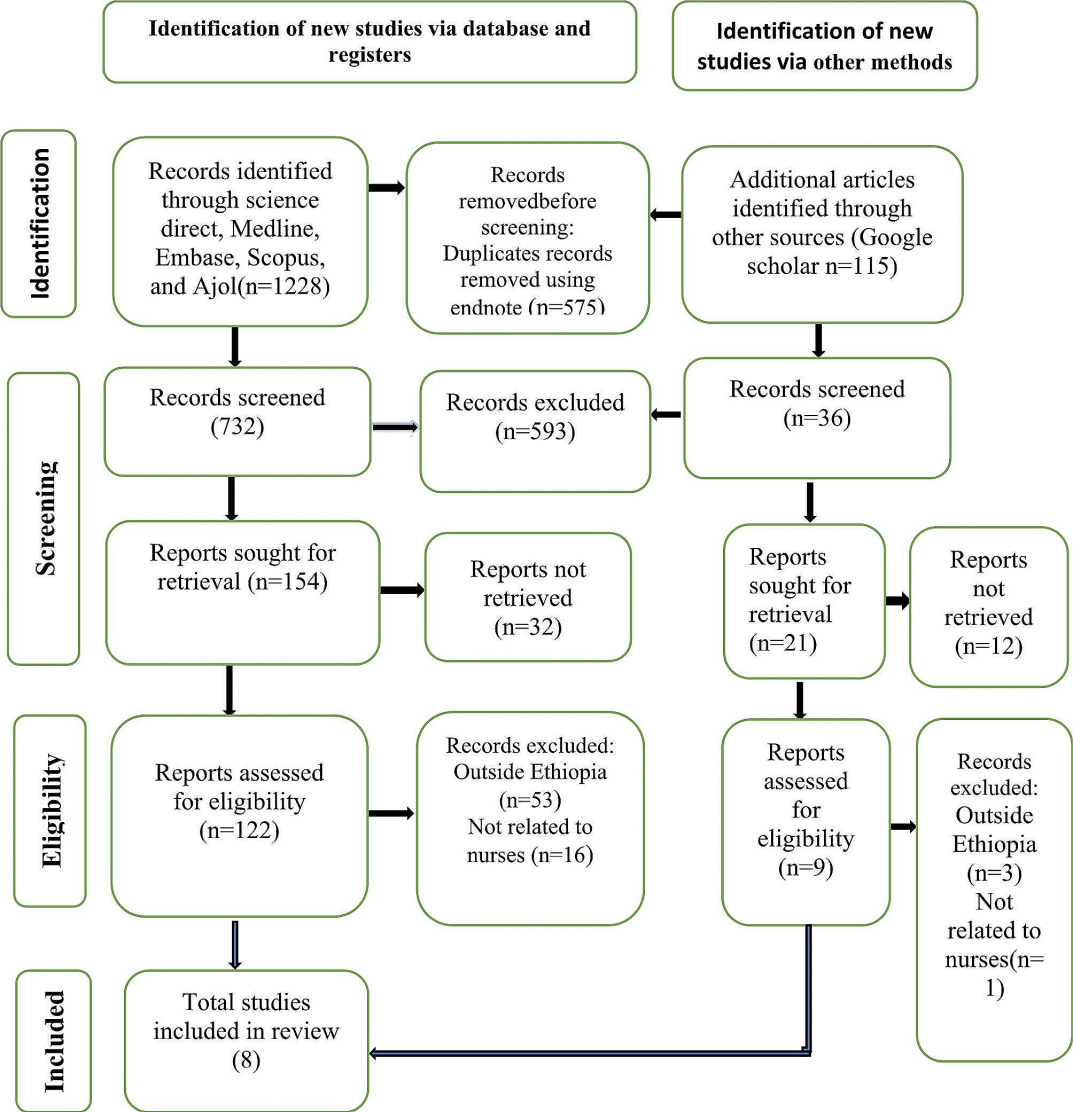
PRISMA flow diagram of the selection process of studies on turnover intention among nurses in Ethiopia, 2024

After conducting the initial comprehensive search concerning turnover intention among nurses through Medline, Cochran Library, Web of Science, Embase, Ajol, Google Scholar, and other sources, a total of 1343 articles were retrieved. Of which 575 were removed due to duplication. Five hundred ninety-three articles were removed from the remaining 768 articles by title and abstract. Following theses, 44 articles which cannot be retrieved were removed. Finally, from the remaining 131 articles, 8 articles with a total 3033 nurses were included in the systematic review and meta-analysis (Fig. 1).

### Study characteristics

All included 8 studies had a cross-sectional design and of which, 2 were from Tigray region, 2 were from Addis Ababa(Capital), 1 from south region, 1 from Amhara region, 1 from Sidama region, and 1 was multiregional and Nationwide. The prevalence of turnover intention among nurses ‘ranges from 30.6 to 80.6%. Table 2.

**Table 2:** Characteristics of studies included in the systematic review and meta-analysis on turnover intention among nurses in Ethiopia, 2024

Name of author	Year	Region	Health facility name	Study design	Sample size	Proportion of turnover intention % (95%CI)
Asegid A et al.	2014	Sidama	Sidama zone public hospitals	Cross-sectional	278	30.6% (95% CI = (25–36)
Getie G.A et al.	2013	Amhara	Public hospitals in east Gojam zone	Cross-sectional	372	59.4% (95% CI: 54.4–64.3)
Ayalew F et al.	2015	Ehiopia	Nationwide	Cross-sectional	425	50.2% (95% CI= (45.4–54.9)
Getachew N et al.	2023	South	Governmental hospitals in southern Ethiopia	Cross-sectional	384	39.8% (95% CI:34.9–44.6)
Wubetie A et al.	2020	Addis Ababa	Governmental hospitals in Addis Ababa	Cross-sectional	102	77.59% (95% CI: (1.03, 50%)
Woldekirkos et al.	2020	Addis Ababa	Federal hospitals in Addis Ababa	Cross-sectional	408	80.6 (95% CI:76.7, 84.4)
Negarandeh R et al.	2020	Tigray	Tigray region	Cross-sectional	634	43.9% (95%CI: 40, 47.7)
Gebregziabher D	2023	Tigray	Axum comprehensive and specialized hospital	Cross-sectional	608	64.9% ((95% CI: 57.2%, 72.5%)

### Pooled prevalence of turnover intention among nurses in Ethiopia

**Fig. 2:**
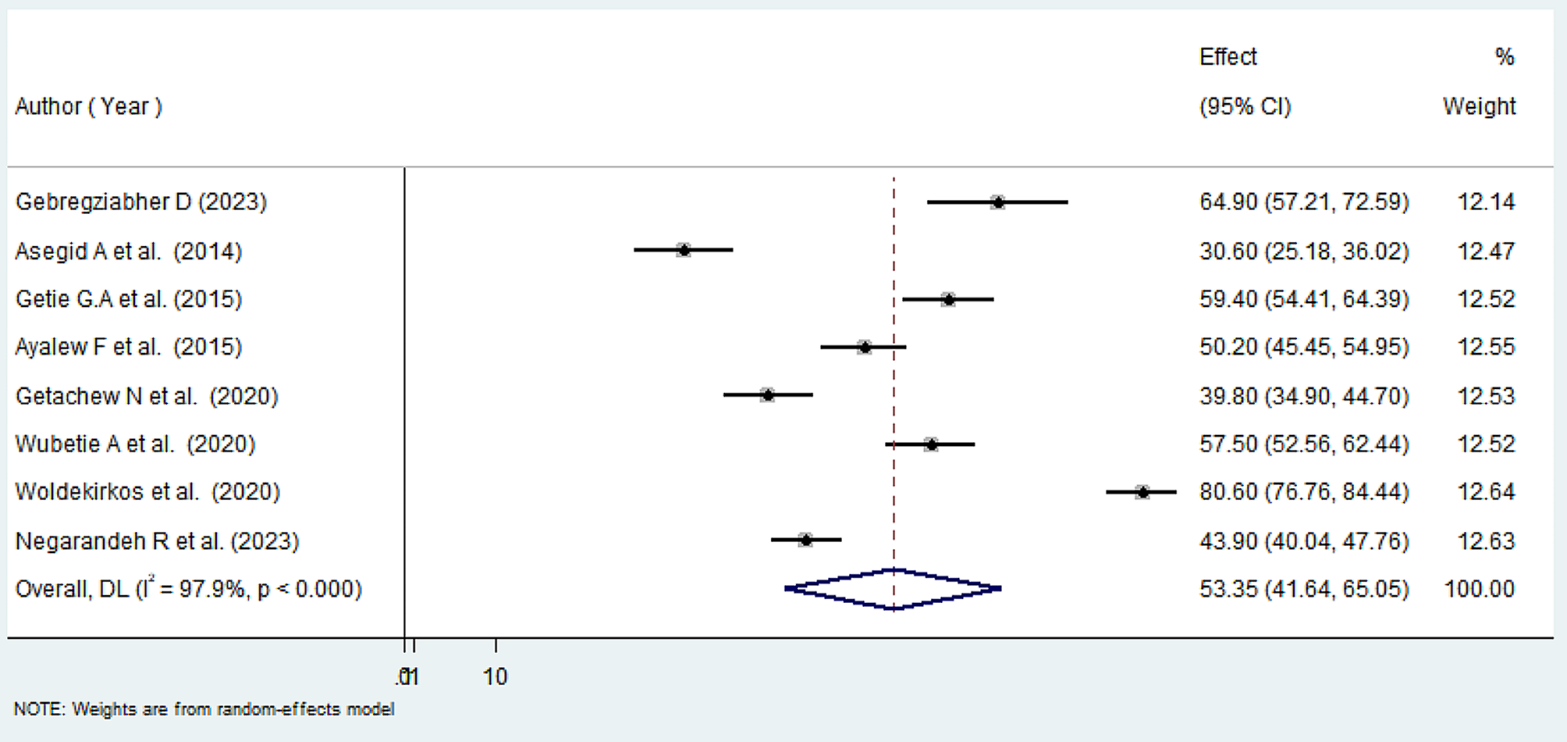
Forest plot showing the pooled proportion of turnover intention among nurses in Ethiopia, 2024

Our comprehensive meta-analysis revealed a notable turnover intention rate of 53.35% (95% CI: 41.64, 65.05%) among Ethiopian nurses, accompanied by substantial heterogeneity between studies (I^2^ = 97.9, *P* = 0.000) as depicted in Fig. 2. Given the observed variability, we employed a random-effects model to analyze the data, ensuring a robust adjustment for the significant heterogeneity across the included studies.

### Subgroup analysis of turnover intention among nurses in Ethiopia

**Fig. 3:**
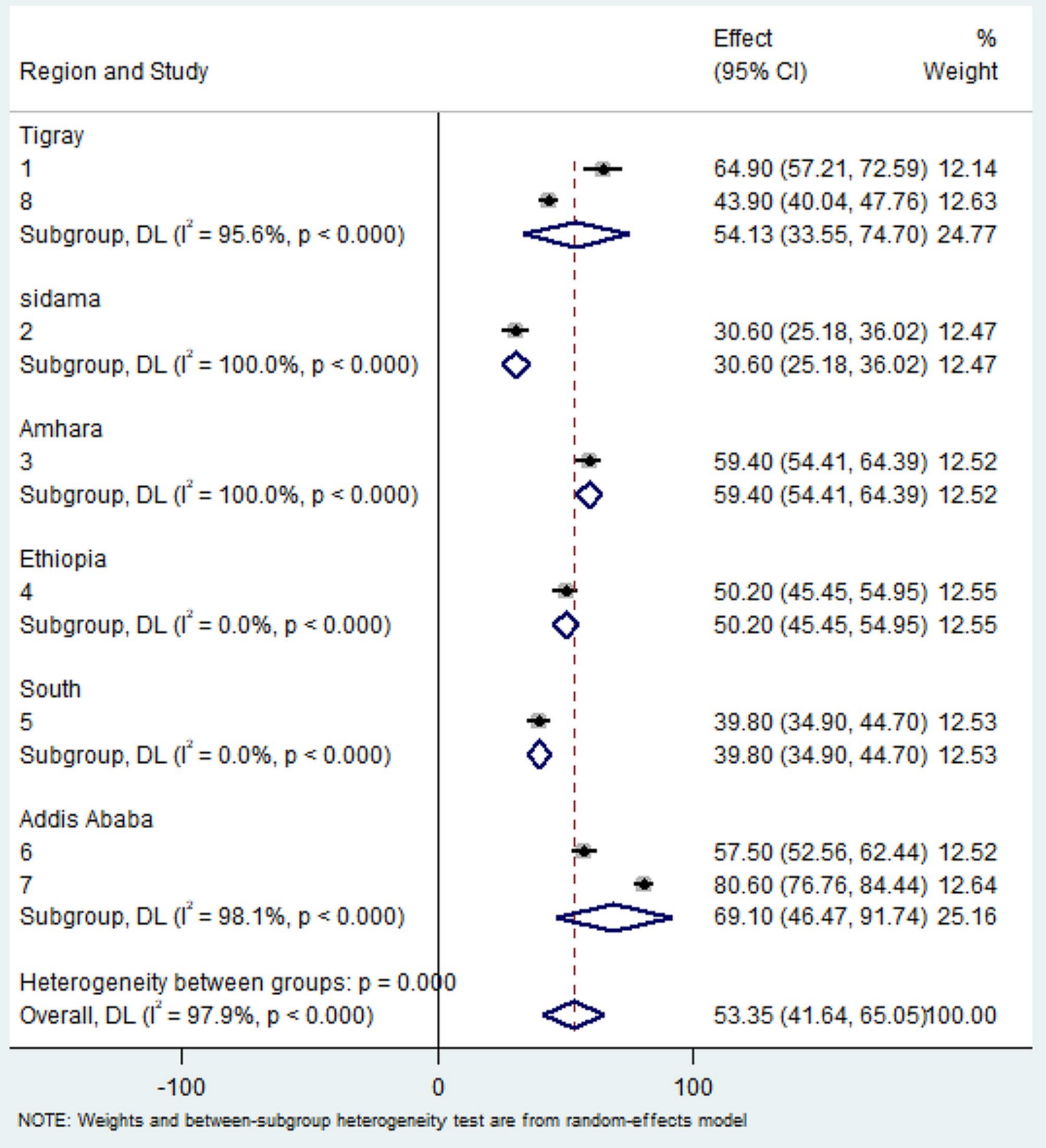
Subgroup analysis of systematic review and meta-analysis by region of turnover intention among nurses in Ethiopia, 2024

To address the observed heterogeneity, we conducted a subgroup analysis based on regions. The results of the subgroup analysis highlighted considerable variations, with the highest level of turnover intention identified in Addis Ababa at 69.10% (95% CI: 46.47, 91.74%) and substantial heterogeneity (I^2^ = 98.1%). Conversely, the Sidama region exhibited the lowest level of turnover intention among nurses at 30.6% (95% CI: 25.18, 36.02%), accompanied by considerable heterogeneity (I^2^ = 100.0%) **(**Fig. 3**).**

### Publication bias of turnover intention among nurses in Ethiopia

**Fig. 4:**
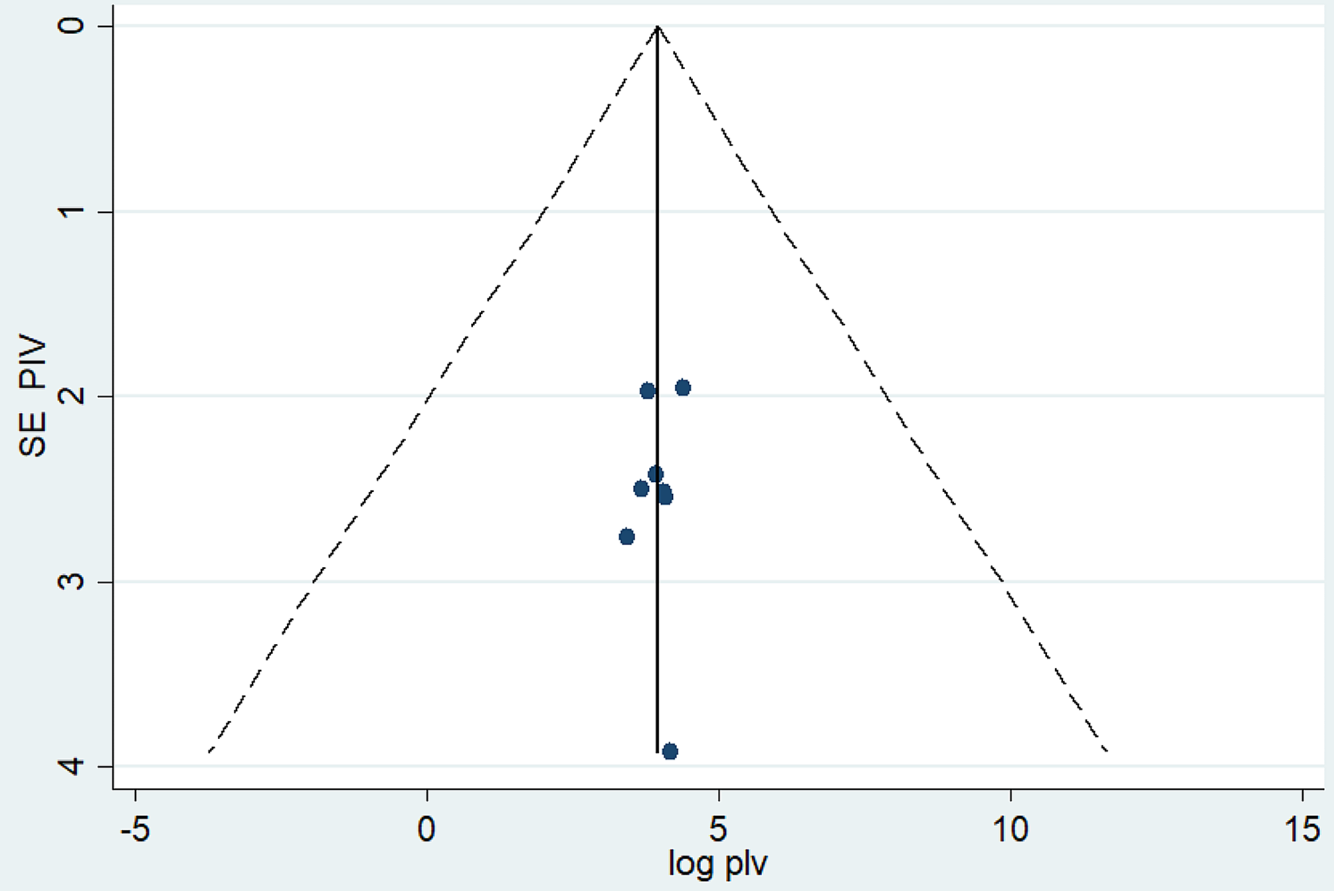
Funnel plot of systematic review and meta-analysis on turnover intention among nurses in Ethiopia, 2024

The Egger’s test result (*p* = 0.64) is not statistically significant, indicating no evidence of publication bias in the meta-analysis (Table 3). Additionally, the symmetrical distribution of included studies in the funnel plot (Fig. 4) confirms the absence of publication bias across studies.

**Table 3:** Egger’s test systematic review and meta-analysis on turnover intention among nurses in Ethiopia, 2024

Standard effect	Coefficient	Standard Error	t	*P* > t	95% CI
Slope	4.255256	0.6262003	6.80	0.000	5.787513
Bias	− 0.1263578	0.2577993	-0.49	0.641	0.5044544

### Sensitivity analysis

**Fig. 5:**
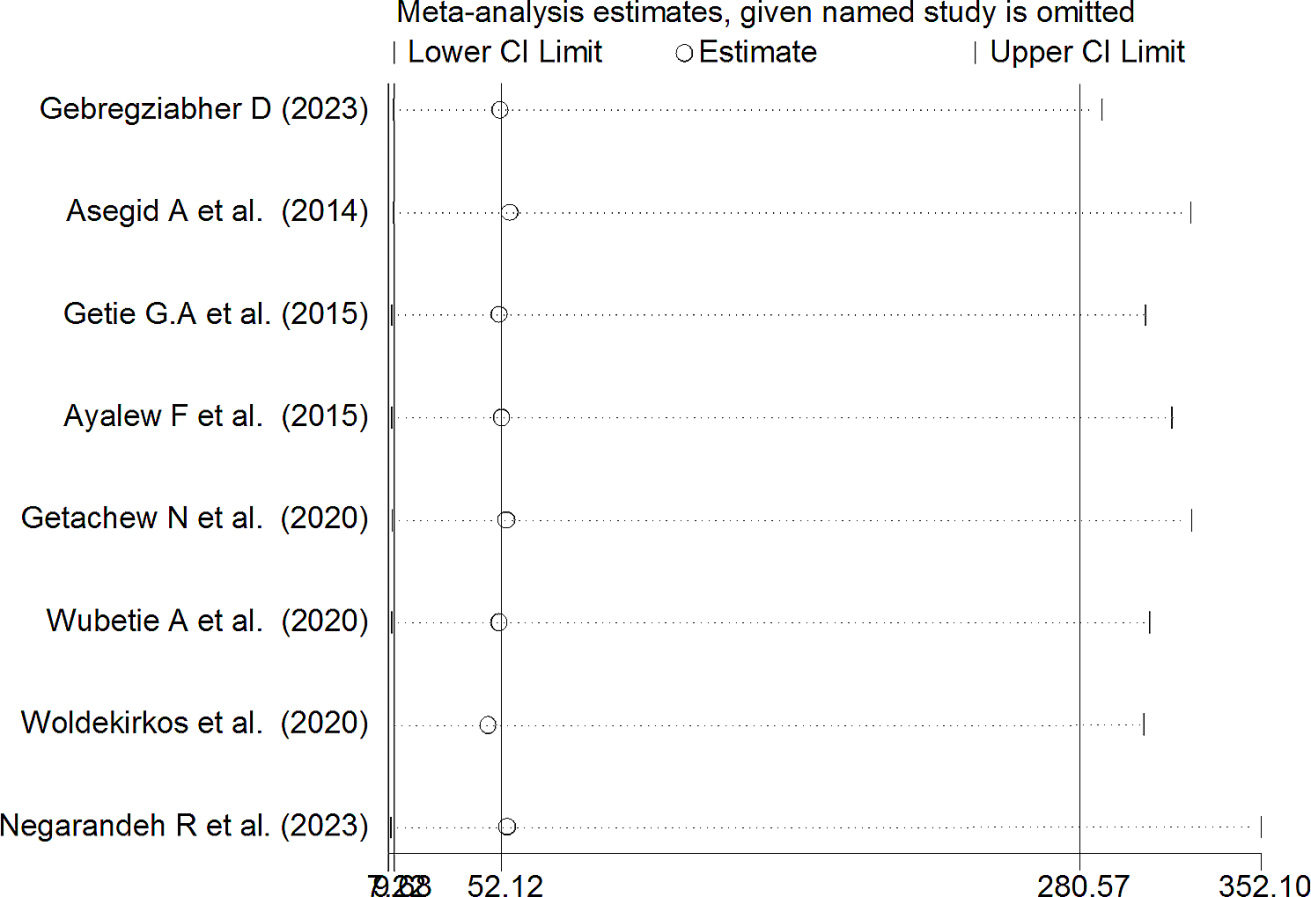
Sensitivity analysis of pooled prevalence for each study being removed at a time for systematic review and meta-analysis of turnover intention among nurses in Ethiopia

The leave-out-one sensitivity analysis served as a meticulous evaluation of the influence of individual studies on the comprehensive pooled prevalence of turnover intention within the context of Ethiopian nurses. In this systematic process, each study was methodically excluded from the analysis one at a time. The outcomes of this meticulous examination indicated that the exclusion of any particular study did not lead to a noteworthy or statistically significant alteration in the overall pooled estimate of turnover intention among nurses in Ethiopia. The findings are visually represented in Fig. 5, illustrating the stability and robustness of the overall pooled estimate even with the removal of specific studies from the analysis.

### Factors associated with turnover intention among nurses in Ethiopia

**Fig. 6:**
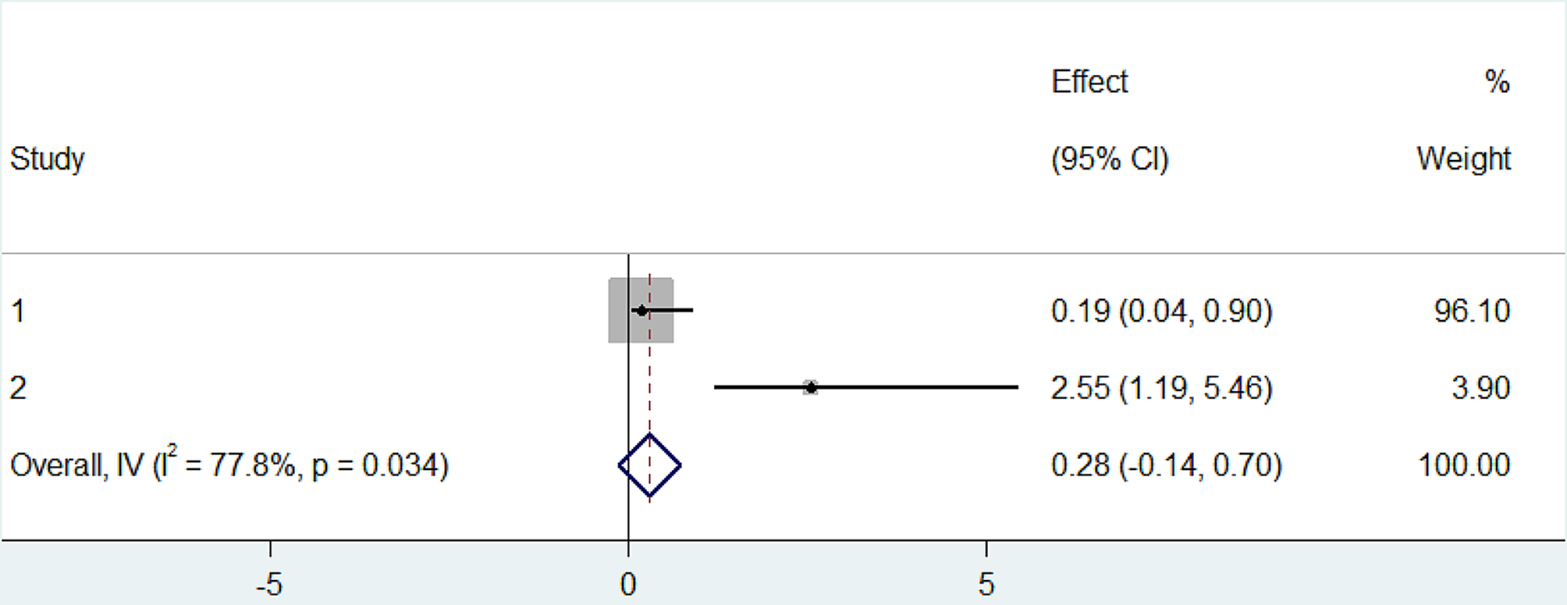
Forest plot of the association between autonomous decision making with turnover intention among nurses in Ethiopia2024

**Fig. 7:**
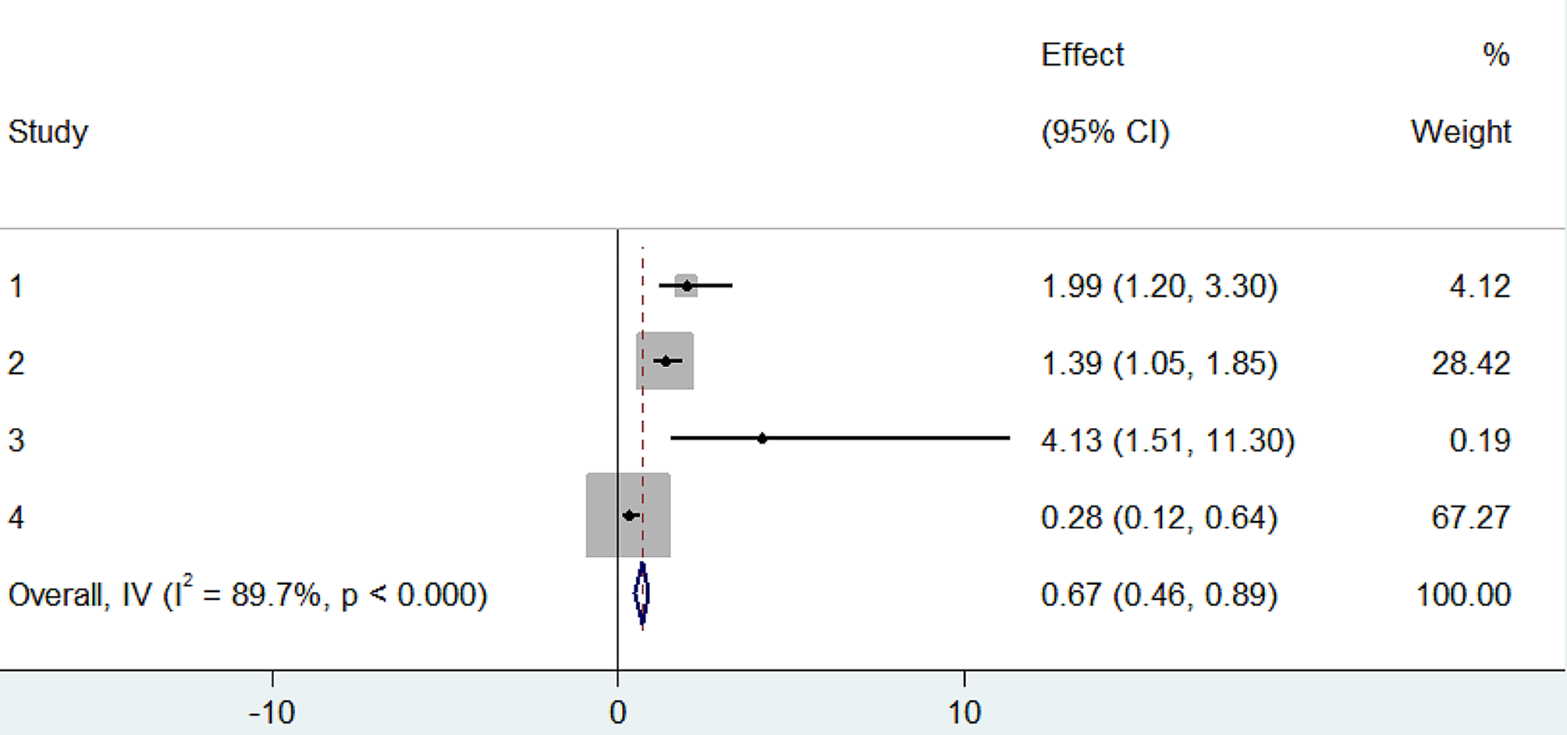
Forest plot of the association between promotion/developpment with turnover intention among nurses in Ethiopia, 2024

In our meta-analysis, we comprehensively reviewed and conducted a meta-analysis on the determinants of turnover intention among nurses in Ethiopia by examining eight relevant studies [6, 29–35]. We identified a significant association between turnover intention with autonomous decision-making (OR: 0.28, CI: 0.14, 0.70) (Fig. 6) and promotion/development (OR: 0.67, CI: 0.46, 0.89) (Fig. 7). In both instances, the odds ratios suggest a negative association, signifying that increased levels of autonomous decision-making and promotion/development were linked to reduced odds of turnover intention.

## Discussion

In our comprehensive meta-analysis exploring turnover intention among nurses in Ethiopia, our findings revealed a pooled proportion of turnover intention at 53.35%. This significant proportion warrants a comparative analysis with turnover rates reported in other global regions. Distinct variations emerge when compared with turnover rates in Alexandria (68%), China (63.88%), and Jordan (60.9%) [5–7]. This comparison highlights that the multifaceted nature of turnover intention, influenced by diverse contextual, cultural, and organizational factors. Conversely, Ethiopia’s turnover rate among nurses contrasts with substantially lower figures reported in Israel (9%) [8], Brazil (21.1%) [9], and Saudi hospitals (26%) [10]. Challenges such as work overload, economic constraints, limited promotional opportunities, lack of recognition, and low job rewards are more prevalent among nurses in Ethiopia, contributing to higher turnover intention compared to their counterparts [7, 29, 36].

The highest turnover intention was observed in Addis Ababa, while Sidama region displayed the lowest turnover intention among nurses, These differences highlight the complexity of turnover intention among Ethiopian nurses, showing the importance of specific interventions in each region to address unique factors and improve nurses’ retention.

Our systematic review and meta-analysis in the Ethiopian nursing context revealed a significant inverse association between turnover intention and autonomous decision-making. The odd of turnover intention is approximately reduced by 72% in employees with autonomous decision-making compared to those without autonomous decision-making. This finding was supported by other similar studies conducted in South Africa, Tanzania, Kenya, and Turkey [37–40].

The significant association of turnover intention with promotion/development in our study underscores the crucial role of career advancement opportunities in alleviating turnover intention among nurses. Specifically, our analysis revealed that individuals with promotion/development had approximately 33% lower odds of turnover intention compared to those without such opportunities. These results emphasize the pivotal influence of organizational support in shaping the professional environment for nurses, providing substantive insights for the formulation of evidence-based strategies targeted at enhancing workforce retention. This finding is in line with former researches conducted in Taiwan, Philippines and Italy [41–43].

## Conclusion

Our meta-analysis on turnover intention among Ethiopian nurses reveals a considerable challenge, with a pooled proportion of 53.35%. Regional variations highlight the necessity for region-specific strategies, with Addis Ababa displaying the highest turnover intention and Sidama region the lowest. A significant inverse association was found between turnover intention with autonomous decision-making and promotion/development. These insights support the formulation of evidence-based strategies and policies to enhance nurse retention, contributing to the overall stability of the Ethiopian healthcare system.

## Recommendations

### Federal ministry of health (FMoH)

The FMoH should consider the regional variations in turnover intention and formulate targeted retention strategies. Investment in professional development opportunities and initiatives to enhance autonomy can be integral components of these strategies.

### Ethiopian nurses association (ENA)

ENA plays a pivotal role in advocating for the welfare of nurses. The association is encouraged to collaborate with healthcare institutions to promote autonomy, create mentorship programs, and advocate for improved working conditions to mitigate turnover intention.

### Healthcare institutions

Hospitals and healthcare facilities should prioritize the provision of career advancement opportunities and recognize the value of professional autonomy in retaining nursing staff. Tailored interventions based on regional variations should be considered.

### Policy makers

Policymakers should review existing healthcare policies to identify areas for improvement in nurse retention. Policy changes that address challenges such as work overload, limited promotional opportunities, and economic constraints can positively impact turnover rates.

### Future research initiatives

Further research exploring the specific factors contributing to turnover intention in different regions of Ethiopia is recommended. Understanding the nuanced challenges faced by nurses in various settings will inform the development of more targeted interventions.

### Strength and limitations

Our systematic review and meta-analysis on nurse turnover intention in Ethiopia present several strengths. The comprehensive inclusion of diverse studies provides a holistic view of the issue, enhancing the generalizability of our findings. The use of a random-effects model accounts for potential heterogeneity, ensuring a more robust and reliable synthesis of data.

However, limitations should be acknowledged. The heterogeneity observed across studies, despite the use of a random-effects model, may impact the precision of the pooled estimate. These considerations should be taken into account when interpreting and applying the results of our analysis.

## Data Availability

Data set used on this analysis will available from corresponding author upon reasonable request.

## Abbreviations

ENA: Ethiopian Nurses Association
FMoH: Federal Ministry of Health
JBI: Joanna Briggs Institute
PRISMA-P: Preferred Reporting Items for Systematic review and Meta-analysis Protocols
